# Urban–rural disparities in fall risk among older Chinese adults: insights from machine learning-based predictive models

**DOI:** 10.3389/fpubh.2025.1597853

**Published:** 2025-05-15

**Authors:** LiHan Lin, XiaoYang Liu, CaiHua Cai, YiKun Zheng, Delong Li, GuoPeng Hu

**Affiliations:** ^1^College of Physical Education, Huaqiao University, Quanzhou, China; ^2^Provincial University Key Laboratory of Sport and Health Science, School of Physical Education and Sport Science, Fujian Normal University, Fuzhou, China; ^3^Department of Cardiology, Fujian Medical University Affiliated First Quanzhou Hospital, Quanzhou, China

**Keywords:** older people, fall risk, machine learning, rural–urban difference, aging, public health

## Abstract

**Background:**

Falls among older adults are a significant challenge to global healthy aging. Identifying key factors and differences in fall risks, along with developing predictive models, is essential for differentiated and precise interventions in China’s urban and rural older populations.

**Methods:**

The data of 5,876 older adults were obtained from the China Health and Retirement Longitudinal Survey (Waves 2015 and 2018). A total of 87 baseline input variables were considered as candidate features. Predictive models for fall risk over the next 3 years among urban and rural older populations were developed using five machine learning algorithms. Logistic regression analysis was employed to identify key factors influencing falls in these populations.

**Results:**

The fall incidence among older adults was 22.4%, with 23.2% in rural areas and 20.9% in urban areas. Common risk factors across both settings include gender, age, fall history, sleep duration, activities of daily living questionnaire scores, memory status, and chair stand test time. In rural areas, additional risks include being unmarried, having diabetes, heart disease, memory-related medication use, and living in houses built 6–20 years ago. For urban, liver disease, arthritis, physical disabilities, depressive symptoms, weak hand strength, poor relations with children, and digestive medication use are significant risk factors while living in a tidy environment is protective. Random Forest models achieved the highest AUC-ROC and sensitivity in both rural (AUC = 0.732, 95% CI: 0.69–0.78; sensitivity = 0.669) and urban (AUC = 0.734, 95% CI: 0.68–0.79; sensitivity = 0.754) areas. Decision curve analysis confirmed the model’s clinical utility across a range of threshold probabilities. Key predictors included prior experience of falling, gender, and chair stand test performance in rural areas, while in urban areas, experience of falling, gender, and age were the most influential features.

**Conclusion:**

The key factors influencing falls among older people differ between urban and rural areas, and the predictive models effectively identify high-risk populations in both settings. This facilitates targeted prevention and precise interventions, supporting healthy aging in China.

## 1 Introduction

The aging population is a global trend that has intensified concerns over the health of older adults (1). Among various health issues, falls have emerged as a major international public health concern. Studies show that approximately one-third of community-dwelling individuals aged 65 or older and nearly half of those aged 80 or older experience falls annually (2–4). In China, the fall incidence rate among adults aged 60 and above reached 3,799.4 per 100,000 in 2019, resulting in severe outcomes such as head trauma, fractures, and even death, with a mortality rate of 39.2 per 100,000 (5, 6). Fortunately, falls are preventable, and early identification of high-risk individuals is key to reducing fall incidence and the associated healthcare burden (7, 8).

In recent years, machine learning has shown promise in developing accurate fall risk prediction models (9, 10). However, most existing models are based on datasets from developed countries, limiting their applicability to the older population in China due to differences in population characteristics and healthcare infrastructure (11, 12). Current Chinese studies tend to focus on specific clinical populations, rely on conventional statistical methods, and are often limited in geographic scope (13–15). Moreover, the variable selection in these models is often narrow (16, 17), failing to account for a comprehensive range of physical, psychological, behavioral, and environmental risk factors (18, 19).

Importantly, few studies have examined urban–rural disparities in fall risk and its predictors within China. As the world’s largest developing country, China faces substantial urban–rural inequalities in healthcare access, infrastructure, and living conditions (20, 21), all of which may influence fall risk in older adults (22, 23).

To address these gaps, this study draws on nationally representative data from the China Health and Retirement Longitudinal Study (CHARLS, 2015–2018), extracting 87 variables across multiple domains. Using binary logistic regression, we identify key predictors of fall risk over a 3-year period in both urban and rural older adults. We then construct and compare the performance of five machine learning models—logistic regression (LR), support vector machine (SVM), random forest (RF), eXtreme Gradient Boosting (XGBoost), and Light Gradient Boosting Machine (LightGBM). The aim is to provide a reference and theoretical basis for differentiated fall prevention and precise intervention among older adults in China.

## 2 Methods

### 2.1 Data source

CHARLS, conducted by the National School of Development at Peking University, is a comprehensive interdisciplinary survey project. The survey covers 150 counties and 450 communities (villages) across 28 provinces, gathering longitudinal data from a nationally representative sample of individuals over 45 years old through face-to-face household interviews. This data encompasses various dimensions, including socioeconomic status and health conditions, providing a robust foundation for research in aging science. This study was carried out based on data extracted from the CHARLS public database, and all methods were performed according to the relevant guidelines and regulations. Written informed consent was obtained from all participants or their legal agents before the commencement of any study process. The ethics approval for the collection of CHARLS data has been approved by the Peking University Biomedical Ethics Review Committee (IRB00001052-11015). Detailed descriptions, including the sampling procedures, questionnaire, and the raw data used in this study can be accessed at https://charls.pku.edu.cn and the supporting information (https://charls.charlsdata.com/pages/Data/2018-charls-wave4/zh-cn.html).

### 2.2 Study population

**Figure 1:**
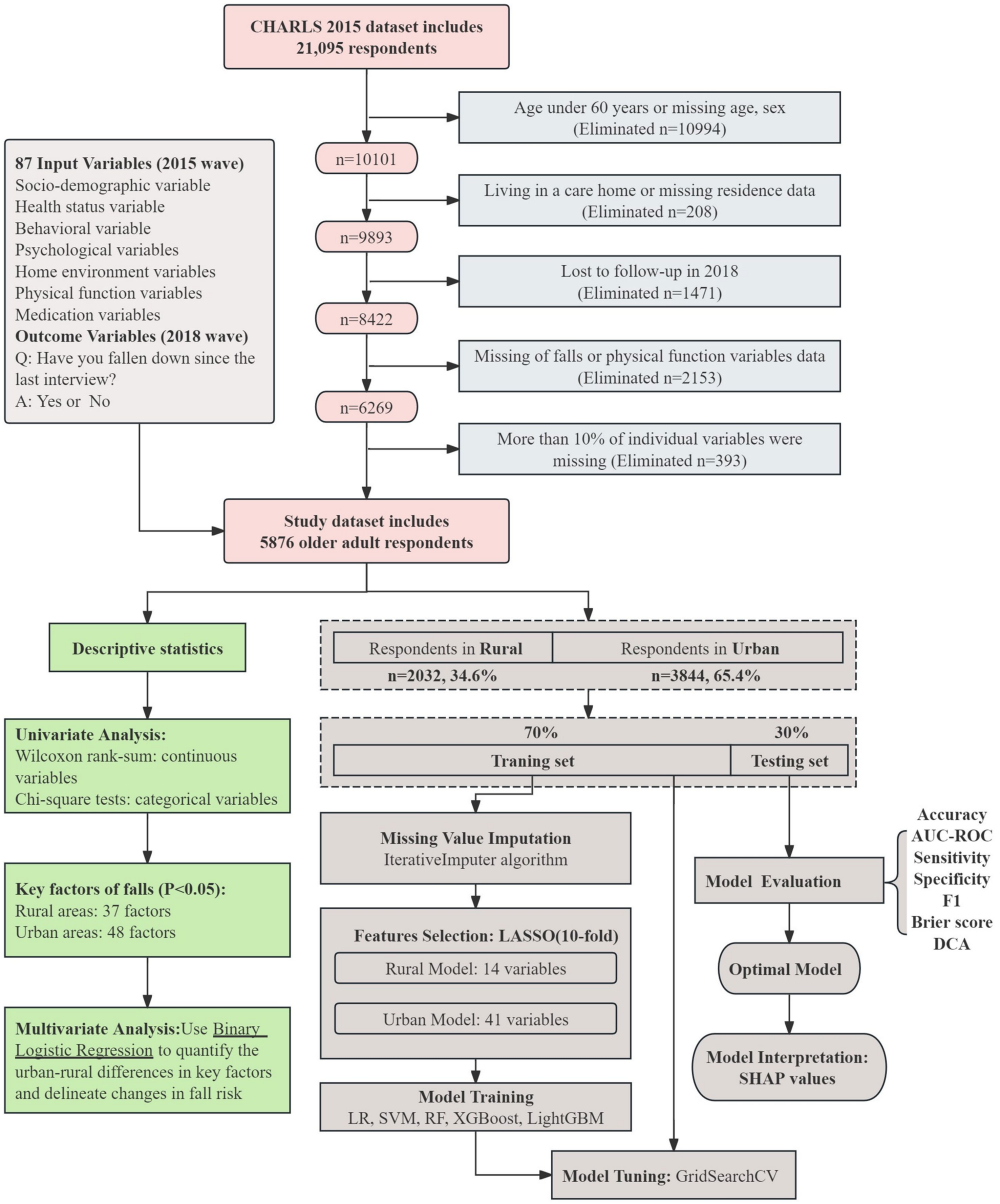
Flowchart of this study. The top section of the figure presents the sample selection process from the CHARLS dataset and an overview of the 87 input variables. The green box in the lower left summarizes descriptive and inferential analyses, including univariate testing and logistic regression. The brown section on the right outlines the machine learning workflow, covering data imputation, LASSO feature selection with 10-fold cross-validation, model training and tuning, evaluation using multiple performance metrics (AUC, sensitivity, specificity, F1, Brier score, and DCA), and model interpretation with SHAP values.

This study utilized data from the CHARLS collected between 2015 and 2018, as the 2020 wave was affected by COVID-19, resulting in fewer survey items and lower response rates, as noted in the Supplementary Methods. Based on the study objectives, the inclusion criteria were Chinese individuals aged 60 and above. Participants were excluded if they met any of the following criteria: (1) missing information on gender, age, or residence, (2) living in a care home, (3) did not participate in the 2015 and 2018 follow-ups, (4) incomplete information on falls and physical function, or (5) more than 10% of individual variables were missing. Ultimately, the study included 5,876 older adults in China. The detailed participant selection process is illustrated in Figure 1.

### 2.3 Research variable

#### 2.3.1 Dependent variable

The Dependent variable of the study was the occurrence of falls within the 3 years following the 2015 wave data collection. This data was collected in the 2018 wave based on the question: *“Have you fallen down since the last interview?”.*

#### 2.3.2 Independent variable

This study utilized a literature review and an existing database, excluding variables with over 10% missing data. A total of 87 baseline variables from the 2015 wave were selected and categorized into 7 groups: socio-demographic, health status, behavioral, psychological, home environment, physical function, and medication.

Socio-demographic variables encompass age, gender, income, living alone, child number, residence, occupation, education, and marital status. Health status variables consisted of vision, hearing, memory, experience of falling, number of chronic diseases, and others. Behavioral variables comprised current exercise habits, current alcohol consumption, current smoking, number of social activities, and sleep duration. Psychological variables include depressive symptoms, life satisfaction, health satisfaction, children relation satisfaction, and marital satisfaction. Home environment variables consisted of type of building, structure of building, house temperature, type of toilet and others. Physical function variables included grip strength, balance ability, walking speed and others. Medication variables included physical examination, health insurance, received inpatient care, antihypertensive medicine and others. All variables were assigned as detailed in Appendix Table 1.

### 2.4 Machine learning models

This study rigorously followed the transparent reporting of a multivariable prediction model for individual prognosis or diagnosis process to construct prediction models (24). We selected five machine learning algorithms—LR, SVM, RF, XGBoost, and LightGBM—to build models predicting fall risk over the next 3 years for urban and rural older adults (Figure 1). Detailed descriptions of these algorithms are provided in the Supplementary Methods.

First, we preprocessed the data by excluding outliers and applying the IterativeImputer algorithm for missing value imputation. To account for differences in feature scales, we normalized the dataset prior to using scale-sensitive algorithms, such as LR and SVM, ensuring all features were on a comparable scale. This prevents features with large values from dominating model training and causing instability or convergence issues (25). In contrast, tree-based models (RF, XGBoost and LightGBM) do not require normalization, as their split criteria rely on feature ranking and split points rather than absolute values.

We employed the least absolute shrinkage and selection operator (LASSO) method (26), which was used for feature selection to identify a subset that enhances prediction accuracy. LASSO regularization was chosen because it can effectively handle multicollinearity and eliminate irrelevant or redundant variables by shrinking their coefficients to zero. The original dataset was split into training and testing sets in a 7:3 ratio, ensuring stratification to maintain class balance. Data imputation and feature selection were performed prior to splitting to prevent any information leakage. Five machine learning models (LR, SVM, RF, XGBoost, and LightGBM) were trained on the training set. Hyperparameter optimization was conducted using GridSearchCV with 10-fold cross-validation applied solely on the training data (27, 28). A customized scoring function combining AUC (weighted at 70%) and sensitivity (weighted at 30%) was used to select the optimal hyperparameters. A detailed justification for this weighting strategy is provided in the Supplementary Methods. The final model performance was evaluated on the independent testing set. Finally, model performance was evaluated on the validation set using a comprehensive set of metrics, including accuracy, AUC, sensitivity, specificity, threshold value and brier score (29). AUC is the primary metric, with higher values indicating better model performance (30). Sensitivity is emphasized because it ensures the model effectively identifies individuals at high risk of falls by maximizing true positives, which is crucial for preventing falls in vulnerable populations (31). The threshold value represents the decision boundary for classifying predictions as positive or negative, influencing the balance between sensitivity and specificity. The Brier score evaluates the accuracy of probabilistic predictions by measuring the mean squared error between predicted probabilities and actual outcomes (32). The calibration of the prediction model was determined according to the Brier score, with a smaller score indicating a better fit. Decision Curve Analysis (DCA) (33) was also performed to evaluate the best model’s clinical usefulness across various threshold probabilities. Model interpretation and feature importance scores were calculated and represented via SHapley Additive exPlanations (SHAP) values from the optimal prediction model (34).

### 2.5 Statistical analysis

This study integrates machine learning models with SHAP values to separately identify the influencing factors of fall risks among older adults in rural and urban areas and compare their differences. SHAP values are utilized to enhance the interpretability of the machine learning models by providing instance-level explanations of feature contributions. Additionally, logistic regression analysis is applied to further quantify the associations between key factors and fall risks, using odds ratios (ORs) to illustrate the direction and magnitude of these relationships. This complementary use of logistic regression and machine learning facilitates both robust prediction and a clearer understanding of the identified factors.

The machine learning model is constructed using Python 3.9, while descriptive statistics and correlation analysis are performed using SPSS 26.0. All continuous variables were non-normally distributed and are therefore presented as median and interquartile range. Categorical data are described as frequencies and percentages. Statistical comparisons were performed using the Wilcoxon rank-sum test for continuous variables and the chi-square test for categorical variables. Multivariate analysis was conducted using binary logistic regression, with the research process illustrated in Figure 1.

## 3 Results

### 3.1 Descriptive results

The 2015 CHARLS baseline survey included 20,967 participants. After applying exclusion criteria, the final sample size was 5,876. The median age was 66 years (IQR: 72–63). Of the participants, 3,844 (65.4%) lived in urban areas, 2,961 (50.4%) were female, and 3,247 (55.3%) had not completed primary education. Detailed baseline characteristics are provided in Appendix Table 2.

Over the 3-year follow-up, 1,317 participants (22.4%) experienced falls. The incidence was 23.2% in urban older adults and 20.9% in rural older adults, with urban older adults showing a higher rate (χ^2^ = 4.28, *p* < 0.05). Among rural older adults, significant differences were found across 39 factors, including age, gender, income, living alone, marital status, memory, fall history, self-rated health, balance, sleep duration, walking test, pain, disability, chronic conditions, vision, and hearing (*p* < 0.05). In urban older adults, significant differences were observed across 48 factors (*p* < 0.05). Detailed information is provided in Appendix Table 3.

### 3.2 Key factors associated with falls in rural–urban old adults

The factors with *p* < 0.05 in the rural–urban older adults were separately included in the backward conditional binary logistic regression analysis, and the results are shown in Tables 1, 2.

**Table 1:** Backward conditional logistic regression analysis of fall risk factors (rural).

Variable	Classification	B	SE	OR	95% CI
Gender	Female				
	Male	−0.316	0.120	0.728*	(0.524, 0.748)
Marital status	Married				
	Unmarried	0.380	0.140	1.462*	(1.111, 1.925)
Live alone	No				
	Yes	0.321	0.179	1.378	(0.971, 1.956)
Income	No				
	Yes	0.237	0.125	1.267	(0.993, 1.619)
Diabetes	No				
	Yes	0.312	0.136	1.367*	(1.048, 1.783)
Memory	Good				
	Fair	0.113	0.152	1.120	(0.831, 1.509)
	Bad	0.293	0.154	1.340*	(1.040, 1.853)
Heart diseases	No				
	Yes	0.281	0.136	1.325*	(1.014, 1.731)
Kidney disease	No				
	Yes	0.336	0.197	1.400	(0.951, 2.060)
ADLQ	No				
	Yes	0.374	0.153	1.454*	(1.077, 1.962)
IADLQ	No				
	Yes	0.359	0.148	1.431*	(1.071, 1.912)
Experience of falling	No				
	Yes	1.110	0.134	3.033*	(2.471, 3.520)
Memory-related medicine	No				
	Yes	1.204	0.483	3.334*	(1.295, 8.583)
House year	0–5				
	6–10	0.361	0.164	1.434*	(1.039, 1.980)
	11–20	0.443	0.168	1.557*	(1.120, 2.164)
	21–30	0.158	0.203	1.171	(0.787, 1.741)
	31–40	0.235	0.276	1.264	(0.736, 2.171)
	Over-40	0.454	0.342	1.575	(0.806, 3.077)
Age	–	0.030	0.008	1.030*	(1.013, 1.047)
Sleep duration	–	−0.076	0.030	0.920*	(0.869, 0.975)
Chair stands test	–	0.025	0.012	1.025*	(1.006, 1.059)

^*^ Indicates *p* < 0.05, CI, confidence interval; OR, odds ratio.

**Table 2:** Backward conditional logistic regression analysis of fall risk factors (urban).

Variable	Classification	B	SE	OR	95% CI
Gender	Female				
	Male	−0.469	0.091	0.626*	(0.524, 0.748)
Memory	Good				
	Fair	0.113	0.152	1.120	(0.831, 1.509)
	Bad	0.293	0.154	1.441*	(1.013, 1.813)
Liver disease	No				
	Yes	0.368	0.161	1.445*	(1.054, 1.981)
Arthritis	No				
	Yes	0.221	0.086	1.247*	(1.054, 1.475)
Physical disabilities	No				
	Yes	0.628	0.220	1.874*	(1.218, 2.883)
ADLQ	No				
	Yes	0.210	0.095	1.234*	(1.025, 1.486)
Experience of falling	No				
	Yes	1.081	0.090	2.949*	(2.471, 3.520)
Depressive symptoms	No Depressive				
	Depressive	0.199	0.090	1.220*	(1.023, 1.454)
Children relation satisfaction	Good				
	Fair	0.071	0.086	1.073	(0.906, 1.271)
	Bad	0.492	0.177	1.636*	(1.157, 2.314)
Hand strength	Normal hand strength				
	Weak hand strength	0.313	0.139	1.368*	(1.042, 1.795)
Bathroom facilities	No				
	Yes	−0.150	0.086	0.861	(0.727, 1.019)
Tidiness	Unclear				
	Clear	−0.165	0.084	0.848*	(0.720, 1.000)
House temperature	Hot				
	Neutral	−0.090	0.138	0.914	(0.697, 1.198)
	Cold	0.414	0.231	1.513	(0.961, 2.382)
Digestive medicine	No				
	Yes	0.318	0.105	1.375*	(1.120, 1.689)
Age	–	0.026	0.007	1.026*	(1.013, 1.039)
Number of chronic diseases	–	0.065	0.020	1.067*	(1.026, 1.110)
Sleep duration	–	−0.089	0.018	0.915*	(0.883, 0.948)
Chair stands test	–	0.024	0.010	1.024*	(1.004, 1.044)

^*^ Indicates *p* < 0.05, CI, confidence interval; OR, odds ratio.

The results indicate that gender, age, history of falling, sleep duration, Activities of Daily Living Questionnaire (ADLQ), memory status, and the time required to complete the chair stands test are common significant factors across both settings. Specifically, being male is associated with a reduced fall risk, while a history of falling significantly increases the risk. Increasing age is associated with a higher likelihood of falling, whereas longer sleep duration serves as a protective factor, reducing the risk of falls. Additionally, poor memory increases the fall risk, and positive results on the ADLQ are associated with a higher fall risk. An increase in the time required to complete the chair stands test also indicates a higher risk of falling.

In rural older adults, additional significant risk factors include being unmarried, having diabetes, heart disease, and the use of memory-related medicine. Living in houses built 6–20 years ago is also a significant risk factor.

In urban older adults, liver disease, arthritis, physical disabilities, depressive symptoms, and weak hand strength are important risk factors specific to urban older adults. Poor satisfaction with children’s relations and the use of digestive medicine also increases the fall risk in this population, while living in a clean environment is an important protective factor specific to urban older adults. Additionally, the raised number of chronic diseases further contributes to the risk of falling in urban settings.

### 3.3 Risk prediction models for falls

#### 3.3.1 Feature selection

**Figure 2:**
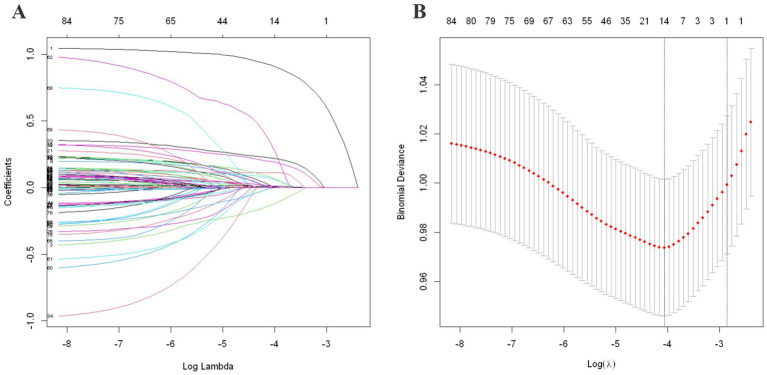
LASSO regularization analysis for rural population. **(A)** Coefficient path by LASSO regularization. **(B)** 10-fold cross-validation curve for *λ* selection. **(A)** Shows the LASSO coefficient path, where each colored line represents the trajectory of a variable’s coefficient as the penalty term (log λ) increases. As λ increases (moving left to right on the x-axis), more coefficients shrink toward zero, enabling variable selection. **(B)** Displays the 10-fold cross-validation curve for selecting the optimal λ. The x-axis shows log-transformed λ values, while the y-axis represents the binomial deviance. The left dotted line indicates the λ with minimum deviance, and the right dotted line indicates the most regularized model within one standard error, which was selected for optimal variable stability.

**Figure 3:**
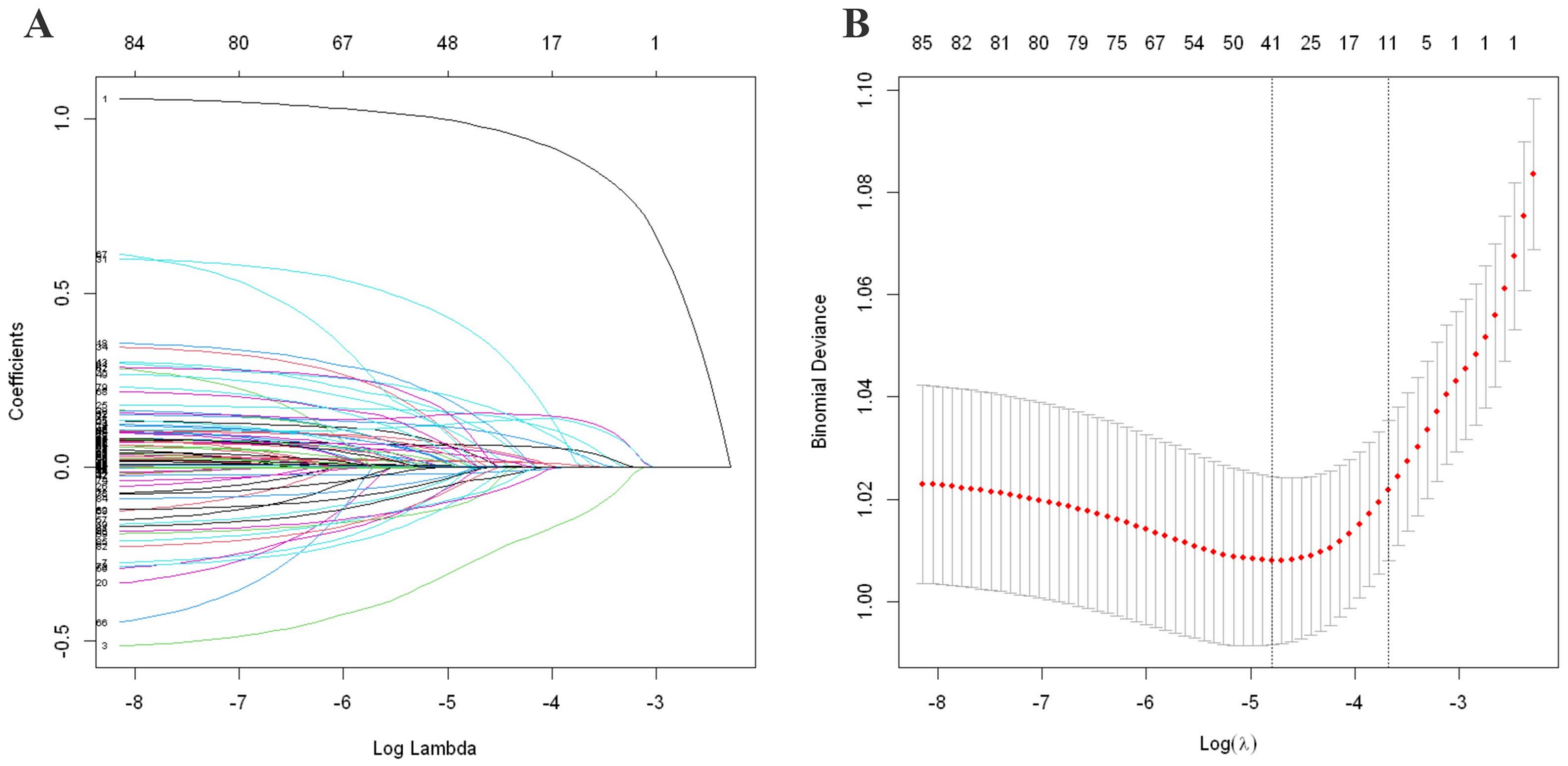
LASSO regularization analysis for urban population. **(A)** Coefficient path by LASSO regularization. **(B)** 10-fold cross-validation curve for λ selection. Similar to Figure 2, **(A)** presents the coefficient shrinkage path across a range of log (λ) values, where coefficients of less relevant variables are progressively reduced to zero. **(B)** Shows the 10-fold cross-validation results, where the λ minimizing binomial deviance is selected as optimal. The one-standard-error rule is also illustrated to indicate a more conservative model with fewer variables, enhancing generalizability.

By applying the LASSO regularization technique, sparsity constraints were imposed to identify the variables with the strongest predictive power. This approach ensures that only the most relevant features are retained, thereby enhancing the model’s accuracy and generalizability. From 87 candidate features, 14 variables for rural and 41 for urban fall prediction were selected. This selection process is visualized in Figures 2, 3 for the rural and urban populations, respectively. The specific variables included in the models are listed in Appendix Table 4.

#### 3.3.2 Model training

The dataset of 5,876 older adults in China was randomly split into a training set (70%) and a testing set (30%) with stratification to preserve class balance. Using the variables selected by LASSO, five machine learning models were developed to predict fall risk among urban and rural older adults over the next 3 years. The hyperparameters of each model were optimized using GridSearchCV with 10-fold cross-validation applied exclusively on the training set. A customized scoring function was implemented to balance both AUC and sensitivity, assigning weights of 0.7 and 0.3, respectively. Each model was retrained using the optimal hyperparameters identified based on the highest combined score. The final model performance was evaluated on the independent testing set. Detailed optimal hyperparameters and their corresponding best combined scores are reported in Appendix Table 5.

#### 3.3.3 Model evaluation

**Figure 4:**
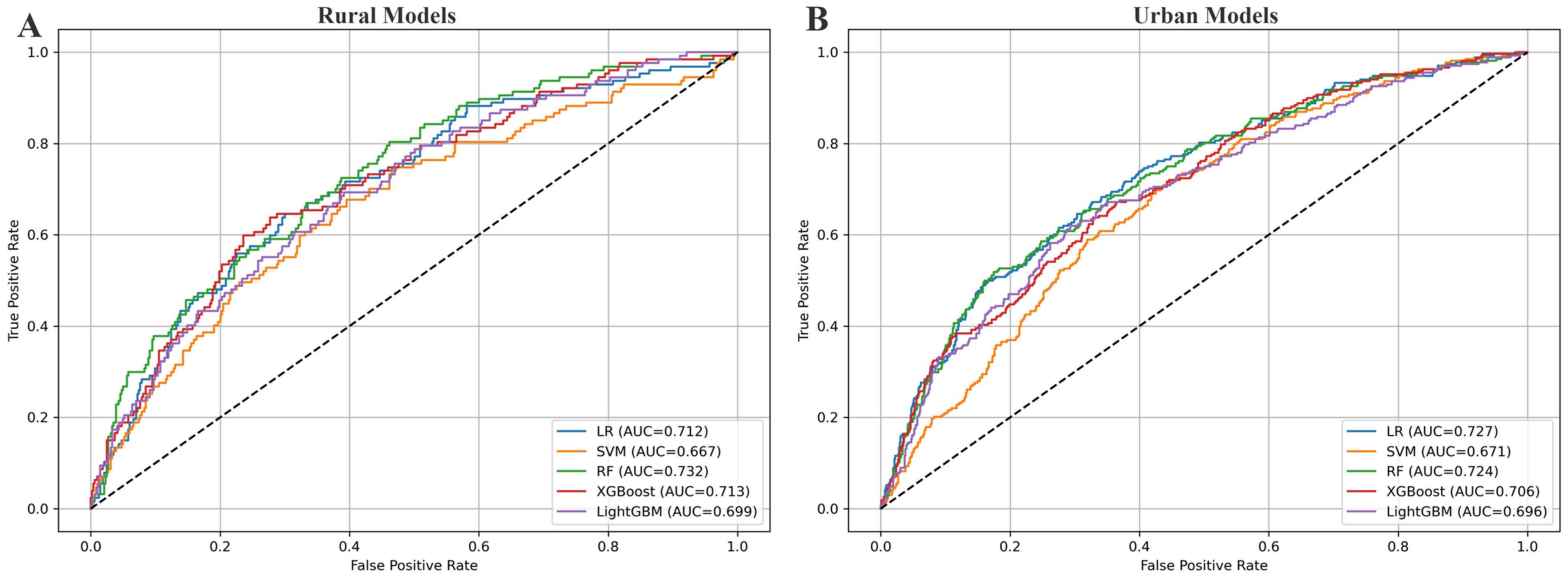
AUC-ROC curves for fall prediction models. This figure presents receiver operating characteristic (ROC) curves for five machine learning models. The x-axis shows the false positive rate (1 – specificity), and the y-axis shows the true positive rate (sensitivity). Each colored line represents a model’s performance, with the area under the curve (AUC) indicating its overall discriminative ability. A curve closer to the top-left corner reflects better classification performance. The dashed diagonal line represents a random classifier with no predictive value (AUC = 0.5). **(A)** Corresponds to the rural sample, and **(B)** to the urban sample.

The optimal parameters were applied to the final models, which were evaluated on the validation set using five metrics: accuracy, AUC-ROC, sensitivity, specificity, and threshold value. The performance of each model is summarized in Table 3. In rural areas, RF showed the best overall performance, achieving an AUC-ROC of 0.732 (95% CI: 0.685–0.782) and a sensitivity of 0.669, indicating a favorable balance between discriminatory power and the ability to identify high-risk individuals. In urban areas, although LR achieved the highest AUC-ROC at 0.727 (95% CI: 0.690–0.758), its sensitivity was relatively low (0.600). Therefore, RF is considered the most appropriate model for predicting falls in urban populations, with a competitive AUC-ROC of 0.724 (95% CI: 0.687–0.757) and a notably higher sensitivity of 0.719. The AUC-ROC curves of the five models for both rural and urban settings are presented in Figure 4.

**Table 3:** Performance of the 5 ML models for predicting falls (rural–urban) on the validation set.

ML models	Threshold	AUC-ROC (95% CI)	Accuracy	Sensitivity	Specificity	F1	Brier score
Rural							
LR	0.209	0.712 (0.662–0.764)	0.689	0.646	0.700	0.463	0.150
SVM	0.196	0.667 (0.609–0.718)	0.620	0.677	0.605	0.426	0.158
RF	0.207	0.732 (0.685–0.782)	0.667	0.669	0.667	0.456	0.150
XGBoost	0.218	0.713 (0.664–0.765)	0.730	0.598	0.764	0.479	0.151
LightGBM	0.197	0.699 (0.650–0.752)	0.626	0.693	0.609	0.436	0.152
Urban							
LR	0.301	0.727 (0.690–0.758)	0.756	0.500	0.834	0.488	0.157
SVM	0.234	0.671 (0.634–0.705)	0.597	0.709	0.563	0.450	0.169
RF	0.271	0.724 (0.687–0.757)	0.754	0.519	0.825	0.495	0.159
XGBoost	0.216	0.706 (0.669–0.738)	0.664	0.638	0.672	0.468	0.162
LightGBM	0.243	0.696 (0.658–0.730)	0.686	0.619	0.707	0.478	0.163

**Figure 5:**
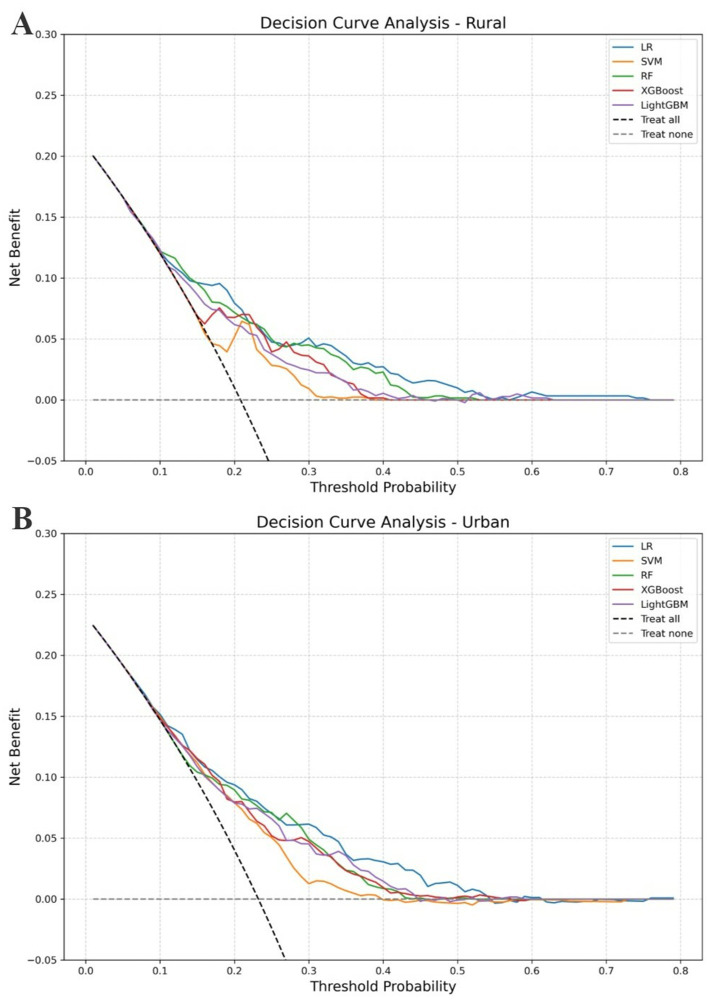
DCA for fall prediction models. **(A)** Rural sample. **(B)** Urban sample. This figure illustrates the net clinical benefit of five prediction models across a range of threshold probabilities. The x-axis represents the threshold probability at which a clinician might choose to intervene (e.g., offer fall prevention measures), and the y-axis shows the corresponding net benefit. Solid colored lines represent different models; higher curves indicate greater clinical utility. The black dashed line (“Treat all”) assumes all individuals are treated, while the gray dashed line (“Treat none”) assumes no one is treated. **(A)** Shows the results for the rural sample, and **(B)** for the urban sample.

To evaluate the clinical utility of the models, we applied DCA, which estimates net benefit across a range of threshold probabilities. As shown in Figure 5, the RF model demonstrated the highest net benefit in rural areas, especially between thresholds of 0.1 and 0.25. LR showed slightly higher net benefit across most thresholds in urban areas, but its lower sensitivity made RF a more balanced and clinically favorable option for identifying high-risk individuals.

#### 3.3.4 Model interpretation

**Figure 6:**
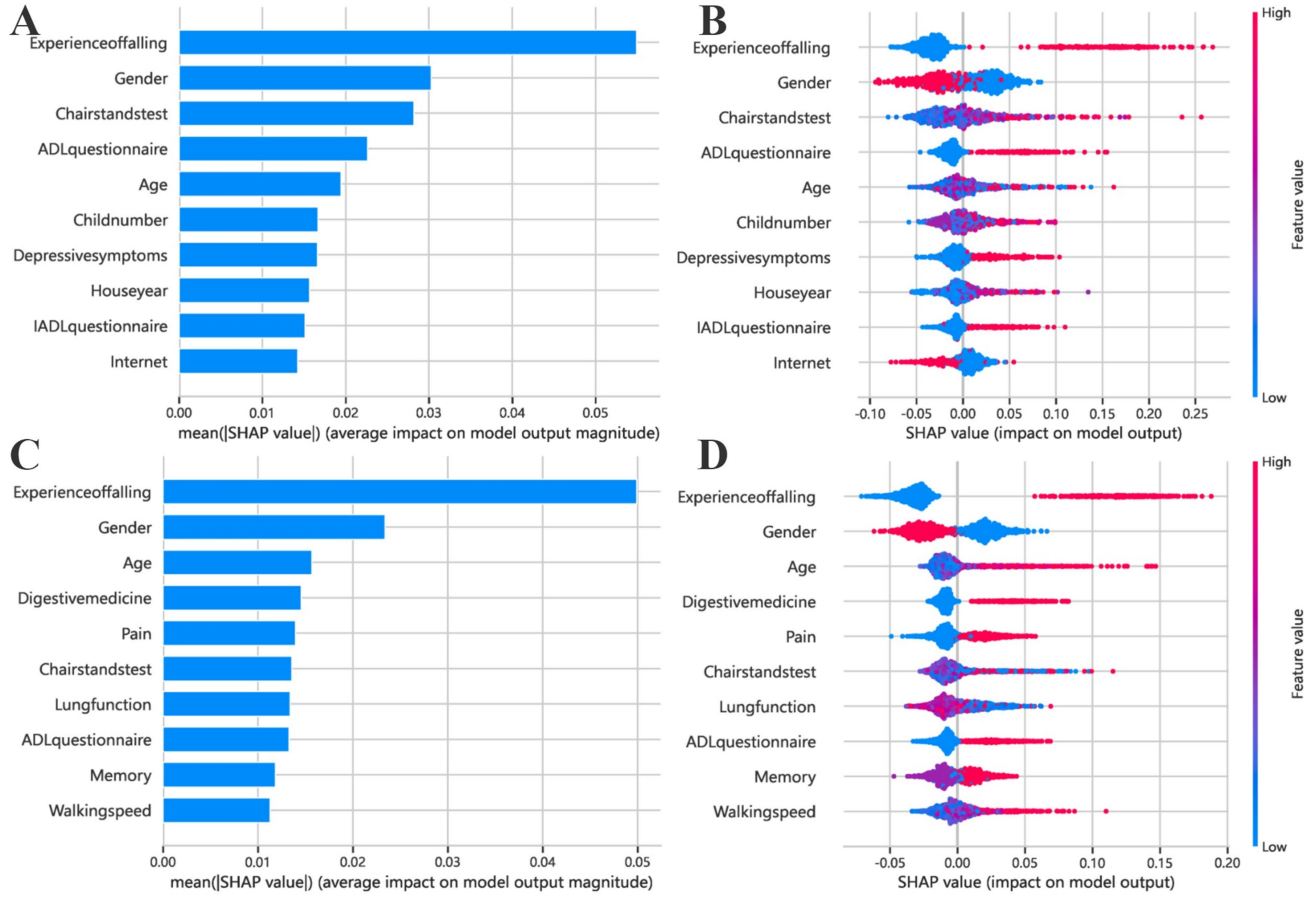
Feature importance of the fall risk prediction model based on SHAP values. **(A,C)** Present the results for rural older adults, while **(B,D)** correspond to urban older adults. Bar plots **(A,B)** display the average magnitude of each feature’s contribution to the model’s prediction, as quantified by the mean absolute SHAP values. Bee swarm plots **(C,D)** provide a more detailed distributional view, where each point represents an individual sample. The x-axis indicates the SHAP value, reflecting the direction and strength of the feature’s impact on the model output (positive values indicate higher fall risk). The y-axis lists the top 10 most important features. Dot colors represent the original feature values, ranging from low (blue) to high (red), allowing visualization of how different feature levels influence predictions. For instance, a cluster of red points on the right suggests that higher values of the corresponding feature are associated with increased fall risk.

SHAP value analysis was conducted to interpret the Random Forest models and quantify the contribution of each predictor to 3-year fall risk among rural and urban older adults, as shown in Figure 6. In both populations, prior experience of falling, gender, age, and chair stand test performance consistently emerged as the most influential features, indicating that individuals who were older, female, had a history of falling, or exhibited poorer lower limb function were at greater risk of future falls. Beyond these shared predictors, distinct features were identified for rural and urban populations. In rural areas, the number of children, depressive symptoms, years of residence, IADL limitations, internet usage, vision, and hypertension were found to significantly influence fall risk. Specifically, greater years of residence (i.e., older housing conditions) and lower internet usage were associated with increased fall risk, while poor vision and hypertension also contributed to higher risk predictions.

In contrast, fall risk in the urban population was more strongly influenced by physical and chronic health indicators, including waist circumference, digestive medication use, lung function, BMI, memory performance, and walking speed. These findings highlight notable differences in fall risk profiles between rural and urban older adults and emphasize the need for setting-specific prevention strategies.

## 4 Discussion

In this study, five machine learning models were developed using 87 variables to predict the 3-year risk of falling among Chinese adults aged 60 years and older in both rural and urban areas. Random Forest achieved the highest performance in both settings, with an AUC-ROC of 0.732 (95% CI: 0.69–0.78) and sensitivity of 0.669 in rural areas, and an AUC-ROC of 0.734 (95% CI: 0.68–0.79) and sensitivity of 0.754 in urban areas. SHAP analysis revealed that while fall history and gender were consistently important in both settings, chair stand test performance had greater predictive value in rural areas, whereas age and digestive medication use were more influential in urban areas. Logistic regression further highlighted common risk factors: gender, age, fall history, sleep duration, ADLQ scores, memory status, and chair stand test time. Unique risk factors for rural older adults included being unmarried (including widowed, divorced, or separated), having diabetes, heart disease, and living in older houses, which were built 6–20 years ago (as of 2015). For urban older adults, they included liver disease, arthritis, physical disabilities, depressive symptoms, weak hand grip strength, poor child relationships, and the use of gastrointestinal medications. Then, a clean living environment was a notable protective factor for them.

In our study, 22.4% of older adults experienced falls over the 3-year follow-up, with a higher incidence in urban areas (23.2%) compared to rural areas (20.9%) (χ^2^ = 4.28, *p* < 0.05). This is slightly lower than the global estimates reported by the World Health Organization, which indicate that approximately 28–35% of people aged 65 years and older fall each year (35). However, it is comparable to previous studies on older Chinese adults, where reported fall prevalence ranges from 14.7 to 34% (19, 36, 37).

Our model’s predictive performance is generally comparable to previous fall risk prediction models among older Chinese adults. Prior studies, employing both traditional statistical methods and machine learning approaches, have reported AUC-ROC values ranging from 0.644 to 0.739 (36, 38), reflecting moderate to good discriminative ability. Compared with previous studies such as Liang et al. (36), which relied on cross-sectional data and a limited set of predictors, our study utilized a broader range of 87 baseline variables and three-year follow-up data to construct more robust fall risk prediction models (AUC up to 0.734 vs. 0.644). Another major contribution of this study lies in the development of separate models for urban and rural older adults, explicitly addressing China’s dual urban–rural development context. This stratified approach captures the distinct socioeconomic, environmental, and behavioral determinants of fall risk in each setting, as revealed through multivariable logistic regression and SHAP-based model interpretation. Together, these methods provide complementary insights into both shared and setting-specific risk factors, thereby enhancing the contextual relevance and practical applicability of the models—an aspect largely overlooked in prior research.

In this study, the RF model consistently achieved the best overall performance in both rural and urban subgroups. It outperformed the other models in terms of sensitivity, AUC-ROC, and F1 score, and also demonstrated one of the lowest Brier scores. This indicates that RF not only identified high-risk individuals more effectively—a priority in fall risk screening—but also provided accurate and well-calibrated probability estimates. Its ensemble structure and ability to capture complex interactions make it particularly suitable for heterogeneous health data. In contrast, models such as XGBoost and LightGBM showed relatively good calibration but lower sensitivity. Logistic Regression offered strong interpretability and a low Brier score, but its reduced sensitivity and suboptimal performance in the DCA limited its effectiveness in screening scenarios. SVM exhibited moderate sensitivity but lacked consistency across other metrics.

Compared to traditional statistical approaches, machine learning methods provide greater flexibility in handling multicollinearity, capturing nonlinear relationships, and improving classification performance without assuming linearity. Moreover, our use of SHAP values enhanced the interpretability of the models, allowing for clearer understanding of risk factors at both population and individual levels. These strengths demonstrate the practical value of machine learning in developing context-sensitive tools for fall risk prediction.

This study highlighted common factors influencing falls among older adults, revealing that older women, whether in rural or urban areas, have a significantly higher risk of future falls compared to men. This finding aligns with previous research and is primarily due to the decline in lower limb strength and bone density in women, particularly postmenopausal, which are often lower than in men of the same age (39, 40). These factors directly affect walking and balance, increasing the likelihood of falls. Other shared risk factors across urban and rural settings include age, fall history, ADLQ, and Chair Stand Test performance. As age increases, natural declines in muscle mass, bone density, and joint flexibility occur, all of which are crucial for maintaining balance and preventing falls (41). A history of falls (42), poor performance in the Chair Stand Test (43), and low ADLQ scores (44) reflect underlying issues like reduced lower limb strength, impaired balance, and diminished physical functioning, all of which heighten the risk of future falls. Additionally, older adults with a history of falls may develop a fear of falling, leading to reduced physical activity and further physical decline (45, 46).

This study found that the prevalence of chronic diseases such as diabetes (23.3%) and heart disease (24.2%) are more common among older adults in rural China compared to those in urban areas, consistent with previous research (47–49). The scarcity of medical resources in rural areas, along with a lack of means for treating and monitoring chronic conditions, contributes to the deterioration of physical health among rural older adults, significantly increasing their risk of falls (50). For prevention, timely treatment of chronic diseases in rural populations should be paired with targeted exercise programs to enhance balance and strength. Being unmarried, especially due to widowhood or divorce, is linked to reduced social support, loneliness, and depression, negatively impacting physical health and mobility (51, 52). In rural China, daily life relies heavily on family and spousal support. Unmarried individuals may lack assistance in tasks like farming and household chores, increasing fall risk due to limited resources, unlike their urban counterparts who have better access to social services and healthcare (53). Older homes, especially those built 6–20 years ago in rural areas, often lack modern safety features such as adequate lighting, non-slip flooring, handrails, and elevators. These deficiencies may increase fall risk by impairing visibility, reducing traction, and limiting support during movement. Structural hazards such as uneven floors or steep stairs can further disrupt gait stability and increase the likelihood of trips or slips (54). Prior studies have shown that home environmental factors are significant contributors to falls among older adults, particularly in settings with outdated or poorly maintained housing (55, 56).

Older homes, especially those built 6–20 years ago in rural areas, often lack modern safety features like adequate lighting, non-slip flooring, handrails, and elevators. Structural issues such as uneven floors or steep stairs further increase fall risks. Preventive measures should include home safety assessments and modifications, such as installing grab bars, improving lighting, and removing tripping hazards, to enhance safety for the rural older adults.

For urban older adults, complex outdoor environments—such as uneven pavements, traffic congestion, and poorly designed public infrastructure—pose greater fall risks, especially for those with arthritis, physical disabilities, or weak grip strength (18, 57). Compared to their rural counterparts, they are more likely to encounter crowded streets, multi-level buildings, and busy transportation systems, all of which increase the likelihood of falls. Prior research has shown that such environmental hazards significantly contribute to fall risk in older adults, particularly those with impaired mobility or balance (58, 59). To reduce fall risk, they should engage in strength and balance exercises, use assistive devices, ensure their living spaces are tidy and safe, and take advantage of community and hospital resources like fall prevention programs (60). Depression and poor relationships with children can lead to social isolation and a lack of support, resulting in reduced social engagement and subsequent physical and cognitive decline, thereby increasing the risk of future falls among urban older adults (61, 62). Preventive strategies should include mental health support and interventions that encourage physical activity and social interaction, aiming to strengthen intergenerational relationships and boost physical activity levels. The association between digestive medication use and fall risk may reflect side effects such as dizziness or indicate underlying frailty or comorbidities. This finding is consistent with prior research on polypharmacy (63, 64), which has been associated with increased fall risk in older adults, particularly when medications such as gastrointestinal agents are involved, and warrants further attention in fall prevention efforts.

This is the first study to apply machine learning to a large, comprehensive dataset aimed at exploring differences in fall risk factors between urban and rural areas and developing fall prediction models for older populations in China. However, it has limitations, including incomplete medical data (e.g., electronic health records, medical imaging, clinical indicators) and the inability to infer causality from detected associations. Additionally, the analysis is based solely on data from 2015 to 2018, without considering recent social developments. Furthermore, the predictive performance of the models still has room for improvement, which may be attributed to the absence of detailed physical fitness and activity-related measures. Future studies could enhance model accuracy by incorporating more precise balance assessments, lower limb muscle strength measurements, and functional mobility evaluations.

## 5 Conclusion

The fall prediction models developed in this study enable targeted screening of older adults at risk of falling within the next 3 years in urban and rural China. The identification of both common and residence-based subgroup-specific risk factors highlights the need for tailored interventions. These findings offer a data-driven basis for integrating predictive tools into community-based fall prevention efforts, particularly under China’s “sports-health integration” initiative. Future research should focus on real-world validation and policy translation to support large-scale implementation.

## Data Availability

The original contributions presented in the study are included in the article/Supplementary material, further inquiries can be directed to the corresponding author.

## Supplementary material

Supplementary F ile 1 Supplementary Methods.

Supplementary File 2 The original raw data used in the study.
