# Supplementary material for: Urban–rural disparities in fall risk among older Chinese adults: insights from machine learning-based predictive models
Source: Front Public Health. 2025 May 15;13:1597853. doi: 10.3389/fpubh.2025.1597853 (PMC12121405; doi:10.3389/fpubh.2025.1597853)
Supplement: Supplementary F ile 1 — Supplementary Methods. [file Supplementary_file_1.pdf]

Supplementary Methods

Rationale for Excluding the 2020 CHARLS Wave

The 2020 wave (Wave 5) of the China Health and Retirement Longitudinal Study (CHARLS) was not included in our analysis due to substantial methodological and content-related disruptions resulting from the COVID-19 pandemic. As documented in the Wave 5 User Guide (https://charls.charlsdata.com/pages/Data/2020-charls-wave5/en.html), CHARLS explicitly acknowledged that the pandemic had a profound impact on survey implementation.

First, as noted in Chapter 1 (General Introduction):"In late 2019 and early 2020, there was an outbreak of Covid-19 in China. In order to document the impact of the pandemic on the lives and health of middle-aged and elderly people in China, the information related to Covid-19 was additionally collected in wave 5."

This led to the inclusion of a dedicated COVID-19 module, which extended interview time and consequently reduced the breadth of information collected in other areas. As clarified in Chapter 3 (Survey Content):

"What needs to be pointed out is that, due to the extra interview time added by the new COVID module, less information was collected in some other modules in Wave 5 compared to previous waves. For example, information on siblings was not collected in the family module; additionally, there was less information on health status, health care utilization, health insurance, pension and assets in this survey."

Furthermore, the pandemic altered the data collection methods, introducing inconsistencies with previous waves. According to Chapter 4 (Fieldwork and Response):"In each of previous waves, all interviews were conducted face-to-face CAPI interviews. However, in wave 5, in some cases when the respondent fear of being infected by COVID-19, or when he/she lives in a nursing home where visits are not allowed, the video interview was conducted after it was approved by CHARLS headquarter."

These changes—ranging from content reduction to deviations in interview modality—have implications for data completeness, comparability, and measurement consistency. Therefore, to preserve the integrity and comparability of our analysis across waves, we excluded the 2020 wave and limited our study to pre-pandemic data.

Overview of Model Characteristics and Considerations for Application

In this study, five machine learning algorithms were applied: Logistic Regression (LR), Support Vector Machine (SVM), Random Forest (RF), Extreme Gradient Boosting (XGBoost), and Light Gradient Boosting Machine (LightGBM). Each model has unique computational mechanisms and potential advantages depending on the nature of the input data.

Logistic Regression (LR) is a linear model widely used in binary classification tasks. It is relatively simple,

interpretable, and suitable for datasets where the relationship between variables is approximately linear.

Support Vector Machine (SVM) is effective for both classification and regression in high-dimensional spaces. It seeks an optimal hyperplane to separate classes and can handle nonlinear relationships through kernel functions, although it may require careful tuning of parameters and is sensitive to feature scaling.

Random Forest (RF) is an ensemble method based on decision tree bagging. It tends to be robust to overfitting and works well with datasets that include complex feature interactions or noisy and high-dimensional data. Because it builds multiple trees with random feature subsets, it can handle a wide range of variable types and distributions.

Extreme Gradient Boosting (XGBoost) is a boosting-based method that builds trees sequentially, optimizing residual errors at each stage. It includes regularization terms to control overfitting and is known for handling structured/tabular data effectively. XGBoost is often efficient for problems with strong feature interactions and when a smaller number of influential features carry high predictive value.

LightGBM is another boosting algorithm that uses a leaf-wise tree growth strategy. It is designed for efficiency and speed, particularly in large datasets, and can be advantageous when training with large-scale or sparse data. However, it may be more sensitive to overfitting in certain cases without appropriate regularization.

The relative performance of these models can vary depending on the feature composition, variable types, data complexity, and class balance within each subgroup. For instance, differences in variable richness, distributional characteristics, and interaction effects between urban and rural datasets may influence how well each algorithm captures underlying patterns.

Custom Scoring Function for Hyperparameter Optimization

To identify the optimal hyperparameters for each machine learning model, we adopted a customized scoring function that integrates the area under the receiver operating characteristic curve (AUC) and sensitivity, assigning a weight of 70% to AUC and 30% to sensitivity. This approach was designed to balance the need for strong overall classification performance with the clinical necessity of identifying high-risk individuals who may experience a fall.

AUC was selected as the primary component because it reflects the model's ability to distinguish between fallers and non-fallers across all possible thresholds, which is especially useful for evaluating classifiers on imbalanced datasets (1). However, in public health scenarios such as fall prevention—where the cost of false negatives (i.e., failing to identify someone who is truly at high risk) is high—sensitivity becomes critically important (2). Failing to detect at-risk older adults may lead to severe outcomes such as fractures, disability, and even mortality,

particularly when early intervention is possible. Therefore, the scoring function places a moderate emphasis on sensitivity to avoid underestimating high-risk cases.

Prior research in the context of disease screening and prognosis prediction—such as in cancer, diabetes, and geriatric care—has supported the use of composite metrics that incorporate sensitivity into model selection and evaluation (3). These studies highlight the importance of balancing overall model performance with the practical need for early risk detection, especially when working with clinical or public health datasets that feature class imbalance or unequal error costs. Based on these considerations, we weighted AUC at 70% to reflect model discrimination and sensitivity at 30% to emphasize correct identification of true positives. This tailored approach aims to enhance the real-world utility of the predictive models for informing fall prevention strategies in clinical and community settings.

Reference:

[1] Saito, T., and Rehmsmeier, M. (2015). The precision-recall plot is more informative than the ROC plot when evaluating binary classifiers on imbalanced datasets. PLoS One 10, e0118432. doi:10.1371/journal.pone.0118432

[2] Yap, C.W. (2023). Practical considerations for choosing evaluation metrics in healthcare machine learning. J Biomed Inform 136, 104288. doi:10.1016/j.jbi.2022.104288

[3] Kourou, K., Exarchos, T.P., Exarchos, K.P., Karamouzis, M.V., and Fotiadis, D.I. (2015). Machine learning applications in cancer prognosis and prediction. Comput Struct Biotechnol J 13, 8–17. doi:10.1016/j.csbj.2014.11.005
